# Simplified Multireference Coupled‐Cluster Methods: Hybrid Approaches With Averaged Coupled Pair Theories

**DOI:** 10.1002/jcc.70020

**Published:** 2025-01-22

**Authors:** Alexander Waigum, Sarah Suchaneck, Andreas Köhn

**Affiliations:** ^1^ Institute for Theoretical Chemistry University of Stuttgart Stuttgart Germany

**Keywords:** AQCC, CEPA, coupled‐cluster theory, multireference, size‐consistency

## Abstract

We define an approximation to the internally contracted multireference coupled‐cluster method with single and double excitations by a hybrid approach. The rationale is to treat the external pair energy contributions by the coupled‐cluster method, which provides accurate results for a large part of the correlation energy while being tractable as the involved pair cluster operators commute. For the internal and semi‐internal contributions, for which the coupled‐cluster part becomes involved due to non‐commuting operators, a linearized approach based on the coupled‐electron pair approximation (CEPA) is used. For the latter, the CEPA(0) method, the averaged coupled pair functional (ACPF), the averaged quadratic coupled‐cluster (AQCC) method, and the averaged CEPA method are tested. We test the methods concerning size consistency, potential energy curves for C_2_, N_2_, CN, and O_3_ and for the singlet‐triplet splitting of ortho‐, meta‐, and para‐benzynes. Our results show that AQCC provides the most accurate results and stable performance. The main drawback of the method is that it shows small violations of size consistency.

## Graphical abstract

An approximation to internally‐contracted multireference coupled‐cluster (icMRCC) theory is discussed in which the difficult terms are replaced by the coupled‐electron pair approximation. A hybrid of icMRCC with averaged quadratic coupled‐cluster (AQCC) theory appears particularly promising.

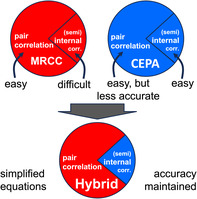

## 1 Introduction

Coupled‐cluster theory [1, 2] is by now established as a standard method in quantum chemistry that provides robust and accurate predictions whenever a single determinant is a good expansion point [3]. However, there are many instances where more than one determinant is required as a reasonable zeroth order description of the system, such as bi‐ or polyradicals or transition metal compounds with partially filled d shells. A generalization of coupled‐cluster theory to these cases is not straightforward, owing to the non‐linear character of its parametrization [4], and the use of configuration interaction methods is still common in these cases [5, 6].

The most straightforward extension to multireference cases, replacing the single reference determinant in the coupled‐cluster ansatz by a multiconfigurational reference, was already foreseen by Čížek [2], but leads to many difficulties as the excitation operators become non‐commuting in this case. Early work in this direction was published in the 1980s by Banerjee and Simons [7, 8]. The method was revived a decade ago in a more general form by Evangelista and Gauss [9] and Hanauer and Köhn [10] and termed internally contracted multireference coupled‐cluster (icMRCC) theory.

One obstacle towards a broader applicability is the complexity of the icMRCC working equations, mainly arising from non‐linear terms of non‐commuting cluster operators. One option to simplify the method is linearization [11] which leads to an interesting connection to the coupled‐electron pair approximation (CEPA) [12, 13, 14] and in fact, icMRCC has a very close connection to the MCCEPA approach of Fink and Staemmler [15]. One drawback of many CEPA variants is their orbital dependence; typically, their definition is based on localized orbitals, which are notoriously difficult to define in a unique way. Alternatively, a number of further CEPA‐like methods were developed that use averaged expressions instead of pair‐dependent shift factors, such as CEPA(0) [14] (also known as linearized coupled‐pair many‐electron theory, L‐CP‐MET [1]), which in the context of multireference calculations was first considered by Laidig and Bartlett [16] (as a linearized multireference coupled‐cluster method) and later by Gdanitz and Ahlrichs along with their work on the averaged coupled pair functional (ACPF) [17], as well as by Ruttink and co‐workers [18]. Analogous methods are the averaged quadratic coupled‐cluster (AQCC) method of Szalay and Bartlett [19, 20] and the averaged coupled‐electron pair approximation (ACEPA) of Ruttink and co‐workers [21]. These methods are essentially minor modifications of the multireference configuration interaction (MRCI) approach and can be implemented rather efficiently. Their accuracy is found to be better than that of MRCI, in particular in the case of MRAQCC [22], but we can expect that it will not fully match that of a full multireference coupled cluster approach.

Recently, Saitow and Yanai proposed a simple approach to merge methods of different accuracy and complexity in a hybrid method [23]. Their rationale was to use an accurate method for all contributions that give a large contribution to the correlation energy but are also straightforward to evaluate, typically the contributions from pair excitations, while other contributions, typically internal and semi‐internal excitations, which give smaller contributions and lead to more difficult equations, are evaluated by a more approximate method. In a recent work [24] we have pointed out that this approach is particularly interesting for icMRCC, for which pair excitations commute and thus lead to much less complex equations than other contributions. In that work, MRCEPA(0) was used for the internal and semi‐internal contributions. The results obtained by this method were promising but not fully satisfactory, as will also become clear from the present work.

Note that the hybrid strategy deviates from the construction principle of embedding schemes [25] or some other hybrid schemes, where a more accurate description for a specific core part is intended, as for instance in the CIPT2 approach of Celani and co‐workers [26]. For a detailed discussion, see Reference [24].

In the present work, we extend on this approach and investigate hybrid approaches that employ CEPA theories with averaged energy shifts like ACPF, AQCC, and ACEPA [21], for internal correlation contributions, merged with icMRCCSD pair energies. In addition, we will test a variant of the hybrid scheme that also treats all one‐body excitations by the coupled‐cluster part. Our main interest is whether the resulting theory is a viable approximation for the full icMRCCSD approach. Test results will therefore be gauged against this method.

In this article, we will first give an overview of our notation and the internally contracted multireference coupled‐cluster theory (Sections 2.1 and 2.2), its relation to the various CEPA‐type approximations (Section 2.3), and how the hybrid methods are constructed (Section 2.4). After some notes on the implementation in Section 3, we will present the results of our test calculations. These are a set of size‐consistency tests (Section 4.1), comparisons of potential energy curves for C_2_, N_2_, CN, and O_3_ (Section 4.2), as well as singlet‐triplet splittings of benzynes (Section 4.3). Conclusions are summarized in Section 5.

## 2 Theory

### 2.1 Basics and Notation

In the following, Ĥ denotes the (non‐relativistic) Born‐Oppenheimer clamped‐nuclei electronic Hamiltonian. The details of its definitions are not relevant for the discussion in this work, but we recall that it contains at most two‐electron interaction terms. As usual for multiconfigurational theories, we will work with a partitioning of the orbital space into closed‐shell orbitals (doubly occupied in all reference configurations), active orbitals (partially occupied), and virtual orbitals (not occupied in any reference configuration). For simplicity, we will use the spin‐orbital formalism in all equations, but we note that spin adaptation is straightforward for the theories considered here and the implementations of the methods will ensure spin‐adapted reference functions and excitations operators in all cases. We will therefore always assume restricted spin‐orbitals, that is, equal spatial orbitals for spin‐up and spin‐down electrons. We adopt the usual index conventions i, j, k, … for closed‐shell spin orbitals, t, u, v, … for active spin orbitals, a, b, c, … for virtual spin orbitals, and p, q, r, …for general spin orbitals (of any of the subspaces). The orbitals are determined in a preceding complete‐active‐space self‐consistent field (CASSCF) computation, and the starting point for all further methods is a CASSCF state of the form

(1) $$\begin{array}{l} |\Psi_{0}\rangle =\underset{\mu }{\sum }|\Phi_{\mu }\rangle c_{\mu } \end{array}$$

where the index μ runs over all configuration state functions. We note that the coefficients $c_{\mu }$ will (optionally) be reoptimized in the methods discussed in the following, but the orbitals remain unchanged. In this work, we will also only consider methods that are invariant with respect to unitary transformations of orbitals within their respective subspace.

Excitation operators for expanding the correlated wavefunctions will be expressed in terms of normal‐ordered creation and annihilation operators ${â}_{q}^{p}=\{{â}_{p}^{†}{â}_{q}\}$, ${â}_{qs}^{pr}=\{{â}_{p}^{†}{â}_{r}^{†}{â}_{s}{â}_{q}\}$, where {·} stands for normal order with respect to the core determinant (the determinant formed from all closed‐shell spin‐orbitals) and ${â}_{p}^{†}$ and ${â}_{p}$ are the usual fermionic creation and annihilation operators, respectively.

### 2.2 Internally Contracted Multireference Coupled‐Cluster Theory

The correlated wavefunction in internally contracted multireference coupled‐cluster (icMRCC) theory is expressed as [9, 10]

(2) $$\begin{array}{l} \left|\Psi_{\text{c}}\rangle =e^{\hat{T}}|\Psi_{0}\right\rangle \end{array}$$

with the cluster operator

(3) $$\begin{array}{l} \hat{T}=\underset{\rho }{\sum }t_{\rho }{\hat{\tau }}_{\rho } \end{array}$$

Here, the index ρ enumerates excitations expressed by the operators ${\hat{\tau }}_{\rho }$, and $t_{\rho }$ are the corresponding amplitudes. In this work, we constrain the excitation operators to single and pair excitations, as summarized in Table 1. In this table, we have also introduced different excitation classes according to the number of electrons excited from the closed‐shell space (‘holes’) and the number of electrons promoted into virtual orbitals (‘particles’). Similar classifications can be found in many other works on multiconfigurational correlation methods, the naming of the spaces as $S_{0}$, $P_{0}$, … was adopted from the work of Celani and Werner [27]. Note that purely active excitations are omitted here; instead, the coefficients $c_{\mu }$ in Equation (1) will be reoptimized, a procedure to which we will refer as ‘reference relaxation,’ see below.

**TABLE 1:** Excitation classes for single excitations ${\hat{\tau }}_{\rho_{1}}$ and double excitations ${\hat{\tau }}_{\rho_{2}}$. The naming of the classes uses the letters I (internal), S (single), and P (pair) according to the number of electrons promoted into the virtual space (‘particles’); the number of electrons excited from the closed‐shell space (‘holes’) is given as subscript.

Class	Holes	Particles	${\hat{\tau }}_{\rho_{1}}$	${\hat{\tau }}_{\rho_{2}}$
$P_{2}$	2	2		${â}_{ij}^{ab}$
$S_{2}$	2	1		${â}_{ij}^{au}$
$I_{2}$	2	0		${â}_{ij}^{uv}$
$P_{1}$	1	2		${â}_{wi}^{ab}$
$S_{1}$	1	1	${â}_{i}^{a}$	${â}_{wi}^{au}$
$I_{1}$	1	0	${â}_{i}^{u}$	${â}_{wi}^{uv}$
$P_{0}$	0	2		${â}_{wx}^{ab}$
$S_{0}$	0	1	${â}_{w}^{a}$	${â}_{wx}^{au}$

Applied to the reference state $\left|\Psi_{0}\right\rangle$, the excitation operators generate internally contracted configurations:

(4) $$\begin{array}{l} \left|\Psi_{\rho }\rangle ={\hat{\tau }}_{\rho }|\Psi_{0}\right\rangle \end{array}$$

The specific property of the coupled‐cluster ansatz, Equation (2), is that it also generates configurations due to higher powers of these operators, which is important to ensure a separable wavefunction and an extensive total energy [3, 4].

The amplitudes $t_{\rho }$ are determined by projecting the Schrödinger equation to the excited configurations

(5) $$\begin{array}{l} \langle \Psi_{\tilde{\rho }}^{\prime }|e^{-\hat{T}}Ĥe^{\hat{T}}|\Psi_{0}\rangle =0 \end{array}$$

where $\left\langle \Psi_{\tilde{\rho }}^{\prime }\right|$ denotes the non‐redundant projection space. That is to say that the initial set of internally contracted configurations ${\hat{\tau }}_{\rho }|\Psi_{0}\rangle$ is not fully linearly independent. The overlap matrix in this space

(6) $$\begin{array}{l} S_{\rho \sigma }=\langle \Psi_{\rho }|\Psi_{\sigma }\rangle \neq \delta_{\rho \sigma } \end{array}$$

is positive semi‐definite but nearly inevitably contains zero eigenvalues. It can be transformed into an orthogonal and non‐redundant basis by

(7) $$\begin{array}{l} |\Psi_{\tilde{\rho }}^{\prime }\rangle ={\hat{\tau }}_{\tilde{\rho }}^{\prime }|\Psi_{0}\rangle =\underset{\sigma }{\sum }{\hat{\tau }}_{\sigma }|\Psi_{0}\rangle X_{\tilde{\rho }}^{\sigma } \end{array}$$

where the transformation matrix $X_{\tilde{\rho }}^{\sigma }$ is derived from the inversion of the square‐root of the overlap matrix on the non‐singular subspace. Choosing the transformation matrix leaves a few degrees of freedom, which we can use to ensure that the transformed operators belonging to different particle ranks are strictly orthogonal, that is $\langle \Psi_{0}|{\hat{\tau }}_{{\tilde{\rho }}_{1}}^{\prime †}{\hat{\tau }}_{{\tilde{\rho }}_{2}}^{\prime }|\Psi_{0}\rangle =0$. While this is trivial in the single‐reference case, this issue requires attention in multireference settings due to the presence of active‐active spectator excitations [10, 28, 29, 30]. As an example, the single and double excitations from the $I_{1}$ subspace have a non‐vanishing overlap:

(8) $$\begin{array}{l} \left\langle \Psi_{0}|{â}_{u\prime }^{i\prime }{â}_{wi}^{uv}|\Psi_{0}\rangle =\delta_{i^{\prime }i}(-\gamma_{wu\prime }^{uv}-\delta_{u\prime }^{u}\gamma_{w}^{v}+\delta_{u\prime }^{v}\gamma_{w}^{u}\right) \end{array}$$

where $\gamma_{w}^{u}=\langle \Psi_{0}|{â}_{w}^{u}|\Psi_{0}\rangle$ and $\gamma_{wx}^{uv}=\langle \Psi_{0}|{â}_{wx}^{uv}|\Psi_{0}\rangle$ are the one‐ and two‐particle reduced density matrices of the reference state. In the orthogonalization scheme developed for icMRCC, we ensure that first the single excitations are orthogonalized, which corresponds (in case of $I_{1}$) to an inversion of the one‐particle hole density ($\delta_{w}^{u}-\gamma_{w}^{u}$). Afterwards, the space of all $I_{1}$ doubly excited configurations is orthogonalized to the configurations reached by the single excitations, and the resulting space is orthogonalized. As a result of this procedure, the orthogonalized double excitations ${\hat{\tau }}_{{\tilde{\rho }}_{2}}^{\prime }$ in the spaces $I_{1}$, $S_{0}$, and $S_{1}$ are linear combinations of the ‘primitive’ two‐ and one‐body excitations, ${\hat{\tau }}_{\rho_{2}}$ and ${\hat{\tau }}_{\rho_{2}}$. For linear theories, this special choice is in principle irrelevant, as the double excitations alone span the entire excitation space (including the singly excited configurations), but for non‐linear theories like icMRCC, this clearly matters [10, 30]. We note that a similar choice was discussed in the context of size‐extensivity of multireference perturbation theories [28].

For formal purposes, we introduce the biorthogonal configuration space

(9) $$\begin{array}{l} \left\langle {\bar{\Psi }}_{\rho }|=\underset{\sigma }{\sum }{\left({\tilde{S}}^{-1}\right)}_{\rho \sigma }\langle \Psi_{\sigma }\right| \end{array}$$

where

(10) $$\begin{array}{l} {\left({\tilde{S}}^{-1}\right)}_{\rho \sigma }=\underset{\tilde{\omega }}{\sum }X_{\tilde{\omega }}^{\rho }X_{\tilde{\omega }}^{\sigma } \end{array}$$

is the pseudo‐inverse of the overlap matrix. Note that the non‐singular subspace is defined for singular values s>η. While for formal discussion any positive η>0 is acceptable, for practical computations with finite precision, η has to be constrained to values on the order $>10^{-6}$.

Projection to the reference space gives another set of equations from which relaxed coefficients $c_{\mu }$ and the updated total energy can be determined:

(11) $$\begin{array}{l} \underset{\nu }{\sum }\langle \Phi_{\mu }|(e^{-\hat{T}}Ĥe^{\hat{T}}-E)|\Phi_{\nu }\rangle c_{\nu }=0 \end{array}$$

For icMRCC computations with ‘reference relaxation,’ Equations (5) and (11) have to be solved simultaneously. The two equations can be combined into a stationary energy functional [10]

(12) $$\begin{array}{c} \begin{array}{ll} & L=\underset{\mu }{\sum }{\bar{c}}_{\mu }\langle \Phi_{\mu }|e^{-\hat{T}}Ĥe^{\hat{T}}|\Psi_{0}\rangle +E\left(\underset{\mu }{\sum }{\bar{c}}_{\mu }\langle \Phi_{\mu }|\Psi_{0}\rangle -1\right)\\ & \;+\underset{\tilde{\rho }}{\sum }\lambda_{\tilde{\rho }}\langle \Psi_{\tilde{\rho }}^{\prime }|e^{-\hat{T}}Ĥe^{\hat{T}}|\Psi_{0}\rangle \; \end{array} \end{array}$$

with Lagrange multipliers ${\bar{c}}_{\mu }$ for the fulfillment of Equation (11) and $\lambda_{\tilde{\rho }}$ for the fulfillment of Equation (5). The energy E appears here as a Lagrange multiplier fixing the norm of the biorthogonal reference function $\left\langle {\bar{\Psi }}_{0}|=\sum_{\mu }{\bar{c}}_{\mu }\langle \Phi_{\mu }\right|$ such that $\langle {\bar{\Psi }}_{0}|\Psi_{0}\rangle =1$. Note that the energy can be computed in two equivalent ways

(13) $$\begin{array}{l} E=\langle \Psi_{0}|e^{-\hat{T}}Ĥe^{\hat{T}}|\Psi_{0}\rangle =\langle {\bar{\Psi }}_{0}|e^{-\hat{T}}Ĥe^{\hat{T}}|\Psi_{0}\rangle \end{array}$$

For practical implementations of the theory, the expansion of the similarity transformed Hamiltonian is an issue. Unlike the single‐reference case, the Baker–Campbell–Hausdorff expansion does not truncate as an operator equation, as not all of the excitation operators commute. The expressions for the energy and the amplitude equations only truncate due to the additional rank exhaustion of the projection manifold; for single and double excitations, this happens for fourfold commutators in case of the energy equations, and eightfold commutators for the amplitude equations [9, 10]. By experience, however, the contributions of higher‐order commutators decay quickly, and keeping only quadratic terms is mostly sufficient [9, 10]. We denote this commutator approximation by $\left(n_{E},n_{A}\right)$ where $n_{E}$ is the rank of commutators kept in the energy expression and $n_{A}$ the rank kept in the amplitude equations. The most often used scheme is the (4,2) approximation, which keeps the exact expression for the energy and truncates after double excitations for the amplitude expressions [10]. Recently, we have also introduced an extension called (4,4s), which introduces all higher‐order commutators that are also present in the single‐reference equations [31]. The reason for this is ensuring direct comparability of single‐reference and multireference results in the limiting case of non‐interacting closed‐shell fragments, but in practice the results from the (4,2) and (4,4s) approximations often differ by only fractions of m*E*_h_ [31].

### 2.3 Coupled‐Electron Pair Theories

As already discussed in Reference [11], there is a close relation between icMRCC and the coupled‐electron pair approximation (CEPA); if the unlinked formalism is invoked, see also Reference [32]. Note, however, that unlike the single‐reference case, the linked and unlinked formalisms are not equivalent in the icMRCC framework [11]. In order to review the main connections, we omit single excitations in the first pass and start with icMRCCD in the unlinked formalism, considering only orders of ${\hat{T}}_{2}$ that also give non‐vanishing contributions in the single‐reference case. The energy expression is then

(14) $$\begin{array}{ll} \left\langle \Psi_{0}|Ĥ+Ĥ{\hat{T}}_{2}|\Psi_{0}\right\rangle & =E\; \end{array}$$

and the amplitude equations become

(15) $$\begin{array}{ll} \left\langle \Psi_{0}|{\hat{\tau }}_{{\tilde{\rho }}_{2}}^{\prime †}(Ĥ+Ĥ{\hat{T}}_{2}+\frac{1}{2}Ĥ{\hat{T}}_{2}^{2})|\Psi_{0}\right\rangle & =E\langle \Psi_{0}|{\hat{\tau }}_{{\tilde{\rho }}_{2}}^{\prime †}{\hat{T}}_{2}|\Psi_{0}\rangle \; \end{array}$$

We can rewrite the energy expression as

(16) $$\begin{array}{l} \langle \Psi_{0}|Ĥ|\Psi_{0}\rangle +\langle \Psi_{0}|Ĥ{\hat{T}}_{2}|\Psi_{0}\rangle =E_{0}+E_{\text{c}} \end{array}$$

to partition it into reference and correlation energy. The amplitude equations can be written as

(17) $$\begin{array}{l} \langle \Psi_{0}|{\hat{\tau }}_{{\tilde{\rho }}_{2}}^{\prime †}(Ĥ-E_{0})(1+{\hat{T}}_{2})|\Psi_{0}\rangle +\langle \Psi_{0}|{\hat{\tau }}_{{\tilde{\rho }}_{2}}^{\prime †}(\hat{X}-E_{\text{c}}){\hat{T}}_{2}|\Psi_{0}\rangle =0 \end{array}$$

where we introduced the operator $\hat{X}=\frac{1}{2}Ĥ{\hat{T}}_{2}$. For this contribution, which introduces a non‐linearity in ${\hat{T}}_{2}$, several approximations can be invoked.

One possibility is to ignore this term, $\hat{X}\approx 0$, which recovers the multireference configuration interaction (MRCI) equations (with internally contracted double excitations):

(18) $$\begin{array}{ll} \left\langle \Psi_{0}|{\hat{\tau }}_{{\tilde{\rho }}_{2}}^{\prime †}(Ĥ-E)(1+\hat{T_{2}})|\Psi_{0}\right\rangle & =0\; \end{array}$$

Note that in the case of linearized theories, we can work with the unprojected version of ${\hat{\tau }}_{\rho_{2}}^{\prime }$ that also generates the singly excited configurations, such that Equation (18) is equivalent to the working equations of the fully internally contracted MRCI method. The MRCI approximation, however, bears the problem that the contribution $E_{\text{c}}\langle \Psi_{0}|{\hat{\tau }}_{{\tilde{\rho }}_{2}}^{\prime †}{\hat{T}}_{2}|\Psi_{0}\rangle$ scales quadratically with the electron number n (as both the correlation energy and the norm of the wavefunction grow with n) and thus leads to non‐extensive energies.

Obviously, the aim has to be that $\left\langle \Psi_{0}|{\hat{\tau }}_{{\tilde{\rho }}_{2}}^{\prime †}(\hat{X}-E_{\text{c}}){\hat{T}}_{2}|\Psi_{0}\right\rangle$ must scale only linearly with the number of correlated electrons. In MRCEPA(0) [16, 17], a full cancellation is assumed, $\hat{X}\approx E_{\text{c}}$, leading to

(19) $$\begin{array}{l} \langle \Psi_{0}|{\hat{\tau }}_{{\tilde{\rho }}_{2}}^{\prime †}(Ĥ-E_{0})(1+{\hat{T}}_{2})|\Psi_{0}\rangle =0 \end{array}$$

This choice leads to significant simplifications in comparison to the full icMRCC theory, while the energy is size‐extensive as long as only pair excitations are involved. This property is lost when also single excitations are introduced, but the errors are rather small [11, 24]. However, MRCEPA(0) has a known tendency to overshoot the correlation energy [16].

Therefore, more elaborate approximations have been developed [12, 13, 15], which, however, still try to avoid the non‐linearity in the cluster operators and approximate this term by a simplified shift term (CEPA shift). Generally, the expressions for the CEPA shift are based on a decomposition of the correlation energy into pair energy contributions

(20) $$\begin{array}{l} E_{\text{c}}=\langle \Psi_{0}|Ĥ{\hat{T}}_{2}|\Psi_{0}\rangle =\underset{K}{\sum }\epsilon_{K} \end{array}$$

where

(21) $$\begin{array}{l} \epsilon_{K}=\underset{\rho \in K}{\sum }\langle \Psi_{0}|Ĥ|\Psi_{\rho }\rangle \langle {\bar{\Psi }}_{\rho }|{\hat{T}}_{2}|\Psi_{0}\rangle \end{array}$$

In this expression, K is meant to single out a class of excitations ρ, which either share a specific pair of closed‐shell or active indices (giving pair energies) or which belong to an entire excitation class, as defined in Table 1. With this, a general CEPA shift expression reads

(22) $$\begin{array}{l} \hat{X}=\underset{KL}{\sum }\underset{\rho \in K}{\sum }|\Psi_{\rho }\rangle U_{L}^{K}\epsilon_{L}\langle {\bar{\Psi }}_{\rho }| \end{array}$$

where the coefficients $U_{L}^{K}$ depend on the different invoked approximations. Orbital invariance is only preserved if classes K involve all orbitals of a given orbital subspace.

A simple choice is $U_{L}^{K}=(n-2)/n$ for all K, L (where, as before, n is the number of correlated electrons) or

(23) $$\begin{array}{l} \hat{X}=\frac{n-2}{n}E_{\text{c}} \end{array}$$

This gives the averaged coupled‐pair functional (ACPF) [17] with the amplitude equations

(24) $$\begin{array}{ll} \left\langle \Psi_{0}|{\hat{\tau }}_{{\tilde{\rho }}_{2}}^{\prime †}(Ĥ-E_{0}+\frac{2}{n}E_{c})(1+{\hat{T}}_{2})|\Psi_{0}\right\rangle & =0\; \end{array}$$

Obviously, the scaling of the shift term is rectified by dividing the correlation energy (which is asymptotically scaling proportional to n) by the number of correlated electrons. Note also that for the limiting case of n=2, the unmodified MRCI equations are recovered, which are exact in the two‐electron case [17].

A similar idea was pursued by Szalay and Bartlett for their multireference Averaged Quadratic Coupled‐Cluster (MRAQCC) method [19, 20, 33]. Here also all $U_{L}^{K}$ are chosen equivalently, giving

(25) $$\begin{array}{l} \hat{X}=\frac{\left(n-2)(n-3\right)}{n(n-1)}E_{\text{c}} \end{array}$$

and resulting in the MRAQCC amplitude equations

(26) $$\begin{array}{ll} \left\langle \Psi_{0}|{\hat{\tau }}_{{\tilde{\rho }}_{2}}^{\prime †}(Ĥ-E_{0}+\frac{2}{n}\frac{\left(2n-3\right)}{\left(n-1\right)}E_{c})(1+{\hat{T}}_{2})|\Psi_{0}\right\rangle & =0\; \end{array}$$

The rationale for the factor in Equation (25) is to partition the correlation energy evenly among all pairs of n correlated electrons by dividing it by (n)(n−1)/2 and to consider for the shift only the pairs of n−2 electrons, giving (n−2)(n−3)/2, to approximately only remove the exclusion principle violating terms [19]. Note that the factor thus vanishes for the two limiting cases of n=2 and n=3.

As Equation (22) suggests, more elaborate schemes are possible. The most elaborate variant for multireference schemes was probably proposed by Fink and Staemmler [15] under the name MCCEPA; their method uses specific shifts for each occupied orbital pair and is specifically designed for localized orbitals. Modified factors for different excitation classes have been suggested both for MRACPF [34] and MRAQCC [33], here we will particularly discuss the MRACEPA functional devised by Ruttink et al. [21]. Note that the MRCEPA(1) method discussed in the same work uses individual pair‐energy‐based contributions for all double excitations from the closed‐shell orbital space, but this method is not considered in the present work.

The scaling factors $U_{L}^{K}$ for MRACEPA, compare Equation (22), are chosen according to the excitation spaces (Table 1), and their values are given in Table 2. For the amplitudes, then the general set of equations, Equation (17), is used. The different factors used in Table 2 are defined as 
(27)

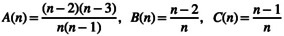

where n can be either the number of correlated closed‐shell electrons $n_{c}$ or correlated active electrons $n_{a}$. Factors A and B are the same as those used for MRAQCC and MRACPF. Note that $U_{L}^{K}$ are chosen symmetric, as also evident from Table 2. For comparison, for MRACPF, all entries in the table are set to $B(n_{c}+n_{a})$, while for MRAQCC all entries are $A(n_{c}+n_{a})$; for MRCEPA(0), all entries are 1, and for MRCI, all entries are 0.

**TABLE 2:** Shift factors $U_{L}^{K}$ used for MRACEPA [21]. The individual factors are defined as in Equation (27), $n_{a}$ is the number of active electrons and $n_{c}$ is the number of correlated closed‐shell electrons.

	L							
K	$S_{0}$	$P_{0}$	$I_{1}$	$S_{1}$	$P_{1}$	$I_{2}$	$S_{2}$	$P_{2}$
$S_{0}$	0	$A(n_{a})$	0	0	$B(n_{a})$	0	0	1
$P_{0}$	$A(n_{a})$	$A(n_{a})$	0	$B(n_{a})$	$B(n_{a})$	0	1	1
$I_{1}$	0	0	0	0	0	$B(n_{c})$	$B(n_{c})$	$B(n_{c})$
$S_{1}$	0	$B(n_{a})$	0	0	$C(n_{c})C(n_{a})$	$B(n_{c})$	$B(n_{c})$	$B(n_{c})$
$P_{1}$	$B(n_{a})$	$B(n_{a})$	0	$C(n_{c})C(n_{a})$	$C(n_{c})C(n_{a})$	$B(n_{c})$	$B(n_{c})$	$B(n_{c})$
$I_{2}$	0	0	$B(n_{c})$	$B(n_{c})$	$B(n_{c})$	$A(n_{c})$	$A(n_{c})$	$A(n_{c})$
$S_{2}$	0	1	$B(n_{c})$	$B(n_{c})$	$B(n_{c})$	$A(n_{c})$	$A(n_{c})$	$A(n_{c})$
$P_{2}$	1	1	$B(n_{c})$	$B(n_{c})$	$B(n_{c})$	$A(n_{c})$	$A(n_{c})$	$A(n_{c})$

For reference relaxation, we consider the equation

(28) $$\begin{array}{l} \underset{\nu }{\sum }\langle \Phi_{\mu }|(Ĥ+Ĥ{\hat{T}}_{2}-E)|\Phi_{\nu }\rangle c_{\nu }=0 \end{array}$$

which is a linearized version of Equation (11). In the literature, MRCEPA(0), MRACPF, MRAQCC, and MRACEPA are mostly formulated for uncontracted CI expansions, which gives additional flexibility. For icMRCC with full non‐linear terms, such an uncontracted formulation is not possible, and we therefore remain in the internally contracted framework but consider reference relaxation whenever possible. In order to keep the nomenclature lean, we will not attach the ‘ic’ prefix to all the methods discussed in this work and continue to speak of MRCEPA(0), and so forth, in the following.

As previously mentioned, for linearized methods, considering the manifold $\left\{{\hat{\tau }}_{\rho_{2}}|\Psi_{0}\rangle \right\}$ (or its orthogonalized, non‐redundant equivalent, respectively) is sufficient to cover the space of singly and doubly excited configurations; therefore, all equations in this section were notated with ${\hat{T}}_{2}$. Equivalent results are obtained if instead the manifold considered for icMRCC is used, with explicit representation of single excitations. In order to combine the theories, as described in Section 2.4, we have to transition to this choice, which in our notation means to replace ${\hat{T}}_{2}$ by $\hat{T}={\hat{T}}_{1}+{\hat{T}}_{2}$ and ${\hat{\tau }}_{2}^{\prime }$ by ${\hat{\tau }}^{\prime }={\hat{\tau }}_{1}^{\prime }+{\hat{\tau }}_{2}^{\prime }$ in the equations given in the present section. In addition, the CEPA‐like amplitude and coefficient equations can be summarized to a stationary energy functional

(29) $$\begin{array}{ll} L & =\underset{\mu }{\sum }{\bar{c}}_{\mu }\langle \Phi_{\mu }|Ĥ(1+\hat{T})|\Psi_{0}\rangle +E\left(\underset{\mu }{\sum }{\bar{c}}_{\mu }\langle \Phi_{\mu }|\Psi_{0}\rangle -1\right)\;\\ & \;+\underset{\tilde{\rho }}{\sum }\lambda_{\tilde{\rho }}\langle \Psi_{\tilde{\rho }}^{\prime }|(Ĥ-E_{0}+\hat{X}-E_{\text{c}})(1+\hat{T})|\Psi_{0}\rangle \; \end{array}$$

which will also be useful in the next section.

### 2.4 Hybrid Schemes

As already indicated in the introduction, the main idea of the hybrid scheme is merging the advantageous properties of two approaches, an accurate and a more approximate one, such that the accuracy is compromised as little as possible while computationally complex parts can be eliminated. The icMRCC method is very much suited for this approach. The main contributions to the correlation energy result from pair excitations, called $P_{0}$, $P_{1}$, and $P_{2}$ in Table 1. The cluster operators of these three classes commute mutually, and the complexity of the terms in the amplitude equations that arise from these operators is not much higher than for single‐reference coupled‐cluster theory. Other operators, such as the $S_{1}$‐type cluster operators, do not commute, neither with operators from the same class nor with those from other excitation classes, and lead to very complex and lengthy equations. At the same time, the contributions to the correlation energy are typically smaller, and it is tempting to introduce approximations for those. A straightforward scheme to achieve this is the hybrid approach [23, 24].

The definition of a hybrid method has basically two ingredients: First, the excitation classes are partitioned into two sets, and second, two methods are chosen and are assigned to the excitation classes. Following the nomenclature of Saitow and Yanai [23], the cluster operator is partitioned into *external* and *internal* contributions

(30) $$\begin{array}{l} \hat{T}={\hat{T}}^{\text{e}}+{\hat{T}}^{\text{i}} \end{array}$$

In the scheme advocated by Saitow and Yanai [23], the external contributions comprise the $P_{0}$, $P_{1}$, and $P_{2}$ excitation classes, while all other classes are considered as internal contributions. In those authors' original work, MRCEPA(0) was used as the more exact method for external contributions (covering most of the excitation energy), and CASPT(2) was used for the internal contributions.

In our recent work, as well as in the present work, the main aim is to approximate the icMRCCSD method. Therefore, we will always consider icMRCCSD as the method for the external contributions while several approaches are tested for the internal contributions. These comprise the methods discussed in Section 2.3, MRCEPA(0), MRACPF, MRAQCC, and MRACEPA.

The hybrid method is then defined by merging the stationary energy functionals of two methods, A and B (see Equations (12) and (29)) according to

(31) $$\begin{array}{l} L_{A/B}=L_{\text{A}}^{\text{e}}+L_{\text{B}}-L_{\text{B}}^{\text{e}} \end{array}$$

where the superscript of $L_{\text{A}}^{\text{e}}$ and $L_{\text{B}}^{\text{e}}$ indicates that this functional is set up with a restriction to using only ${\hat{T}}^{\text{e}}$ operators (and the amplitude equations from projections to the corresponding external manifolds) [24]. The hybrid methods will be denoted by the names MRCC/CEPA(0), MRCC/ACPF, and so forth.

In addition to the partitioning of Saitow and Yanai, we will also consider a set of hybrid methods, in which all single excitation operators ${\hat{\tau }}_{\rho_{1}}$ from $S_{1}$, $I_{1}$, and $S_{0}$ are included in the external part. The rationale is that these operators on the one hand do not significantly increase the complexity of the resulting equations, while the overall method has more contributions that are treated as in the target coupled‐cluster method. Also, single excitations contribute to the incomplete size extensivity of methods like MRCEPA(0) [11] and it is interesting to see if this alternative partitioning is advantageous in this respect. The modified methods will be called MRCC/CEPA(0)‐S, MRCC/ACPF‐S, and so forth.

## 3 Implementation and Computational Setup

The icMRCCSD method is implemented in our GeCCo program [35] and the implementation of the hybrid methods was discussed earlier [24]. In a local version, we have also added the implementations of the MRACPF, MRAQCC, and MRACEPA methods and their hybrids with the icMRCCSD method. In analogy to our previous work [24], the hybrids with icMRCCSD considered at most double commutators for both energy and amplitude equations. The GeCCo program was used in conjunction with the Molpro program package [36, 37] which provided the CASSCF orbitals and one‐ and two‐electron integrals transformed into this orbital basis. The computations employed the cc‐pVTZ basis set of Dunning and co‐workers [38], only for the N_2_ calculations was the def2‐QZVPP basis [39] used. The frozen‐core approximation was used throughout.

## 4 Results and Discussion

### 4.1 Size‐Consistency Tests

One important property of coupled‐cluster theory is the correct scaling of the energy with system size (extensivity), which ensures that the accuracy of the method is also maintained for large systems. This property refers to the thermodynamic limit and is difficult to test, in particular for methods that are designed for molecular computations [40]. The usual resort is to check the size consistency of the method, that is, the consistency of the energies from non‐interacting molecules with the energies from computations for the individual molecules. The result of such a test can be viewed under two different aspects: If molecular interaction energies (or bond energies between molecular fragments) are concerned, exact size consistency is mandatory for using the sum of non‐interacting systems as a reference point; this will in some cases also require a proper separation of the reference state. The other aspect is, as indicated above, a test for how strongly the correlation energies computed by this method will deteriorate for larger systems. In this case, small violations of size consistency may still be acceptable in the sense that the method will give reasonably good correlation energies for all system sizes that conceivably can be treated.

In this work, the following tests were conducted:

a. A C_2_ molecule in the singlet state (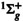) and a non‐interacting neon atom are placed 10,000 $a_{0}$ apart. The 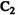 molecule is treated with a full valence active space, CAS(8,8), so all correlated orbitals of 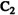 are in the active subspace, while the correlated orbitals of the neon atom are fully in the closed‐shell subspace. This test checks the separability of the active and closed‐shell orbital subspaces for the total correlation energy.
b. The next test uses the same settings as before, but instead the effect of the non‐interacting neon atom on the vertical singlet‐triplet splitting of 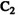 is measured. This test checks the size of non‐separability effects on relative energies.
c. Test (a) is repeated, but for 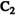, only a minimal active space, CAS(2,2), is employed (consisting of the $2\sigma_{u}$ and $3\sigma_{g}$ orbitals). This test checks the separability of the closed‐shell orbital subspace.
d. The test uses the settings of test (c), but the effect of the non‐interacting Ne atom on relative energy splittings is tested. This test checks non‐separability effects on relative energies in the case of small active spaces.
e. Two singlet 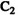 molecules are placed at a far distance (100,000 $a_{0}$), both employing a CAS(2,2) space; for the supersystem, this results in a CAS(4,4). This test checks the size‐consistency in case of the separation of the active shell.
f. Analogous to test (e), but this time two triplet 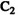 molecules (state 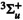) are placed at a far distance (100,000 $a_{0}$), the total spin‐coupled state is a triplet. This test checks the size consistency for entangled states.
g. A singlet 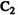 and a singlet 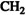 molecule are placed at a far distance (100,000 $a_{0}$). For both molecules again a CAS(2,2) is employed (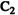 as before; for 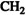, the $a_{1}$ and $b_{1}$ orbitals are chosen, and the molecule is placed in the yz plane). This test is analogous to test (e), but this time the number of correlated electrons is not distributed symmetrically.

We note that the choice of orbitals does not require any special attention for these tests, as the internally contracted approach is fully invariant with respect to orbital rotations within the orbital subspaces (closed, active, virtual) [9, 10].

As some of the methods lead to convergence problems, when reference relaxation is switched on (for more details, see forthcoming sections), we used computations with fixed reference coefficients for these tests. In fact, the reference relaxation contributions are uncritical for size consistency, see the provided data listed in the supporting information Section S1. The results of all tests are summarized in Table 3. We make the following observations:

**TABLE 3:** Size consistency tests for all methods considered in this work, all values are given in kJ/mol. The reported values correspond to the correlation energy missing (for positive sign) in the supersystem of two non‐interacting molecules (as indicated in the column header), except for the $\Delta \Delta_{\text{ST}}$ columns which report the influence of a non‐interacting neon atom on the singlet‐triplet splitting. (S) denotes the molecule in the singlet state, (T) is the molecule in the triplet state, for (S) the state was employed. Reference relaxation was not used for these tests, see text.

Test	(a)	(b)	(c)	(d)	(e)	(f)	(g)
	CAS(8,8)		CAS(2,2)		CAS(2,2)–CAS(2,2)		
Method	C_2_(S)⋯Ne	$\Delta \Delta_{\text{ST}}$	C_2_(S)⋯Ne	$\Delta \Delta_{\text{ST}}$	C_2_(S)⋯C_2_(S)	C_2_(T)⋯C_2_(T)	C_2_(S)⋯CH_2_(S)
MRCCSD	0.000	0.000	0.000	0.000	0.000	0.000	0.000
MRCISD	37.998	0.123	85.775	−4.888	125.797	114.053	66.891
MRCEPA(0)	0.000	0.000	0.000	0.000	0.000	−0.014	0.000
MRACPF	0.735	0.055	−1.299	0.802	0.000	−0.015	−3.770
MRAQCC	2.089	0.091	1.756	0.975	4.642	4.295	−1.892
MRACEPA	0.073	−0.002	2.716	0.129	19.932	13.303	3.925
MRCC/CISD	14.481	−0.516	22.988	−4.751	45.218	34.824	18.163
MRCC/CISD‐S	13.987	−0.719	20.008	−4.293	39.353	30.056	15.749
MRCC/CEPA(0)	0.000	0.000	0.000	0.000	0.000	−0.014	0.000
MRCC/CEPA(0)‐S	0.000	0.000	0.000	0.000	0.000	−0.012	0.000
MRCC/ACPF	1.063	0.006	−0.593	0.411	0.000	−0.015	−2.308
MRCC/ACPF‐S	1.034	−0.020	−0.326	0.313	0.000	−0.013	−1.736
MRCC/AQCC	2.156	−0.008	0.400	0.491	1.974	1.769	−2.004
MRCC/AQCC‐S	2.097	0.054	0.496	0.444	1.620	1.556	−1.564
MRCC/ACEPA	0.064	−0.003	0.782	−0.094	12.823	7.862	1.832
MRCC/ACEPA‐S	0.000	0.000	0.737	−0.068	11.858	7.178	1.503

First of all, the parent method icMRCCSD passes all tests, which is the expected outcome. We note that obtaining this desired behavior involves a proper choice of the amplitude and projection manifold; see References [10, 29, 30], where the most difficult‐to‐achieve property is the separability of entangled open shells [29].

The other extreme is found for the MRCISD method, which is known to not be extensive [17]. The values in Table 3 illustrate the magnitude of size consistency violations. The missing total correlation energy in the supersystem of two molecules can be as large as 100 kJ/mol even for these small systems. Notable non‐separability effects also occur for relative energies, as indicated for the shift of up to 4.9 kJ/mol for the singlet‐triplet splitting by a non‐interacting neon atom.

The CEPA methods strongly reduce the error seen for MRCISD but cannot fully suppress them. The best performance is found for MRCEPA(0), which passes all tests except the one involving entangled spin states. The other CEPA methods show distinctly larger size‐consistency violations. MRACPF is, by construction, very good for symmetrically split systems like the two non‐interacting C_2_ molecules, as it distributes the correlation energy according to the number of correlated electrons. For non‐symmetric systems, however, the error of MRACPF is more similar to those of MRAQCC and MRACEPA. No clear picture arises for MRACEPA. By construction it performs rather well when active and closed‐shell orbital subspaces are separated [tests (a) and (b)], but the errors strongly increase when the closed‐shell orbitals or the active orbitals are distributed among the subsystems. In particular for tests (d) through (f), the errors are significantly larger than those of the more simple MRACPF and MRAQCC methods.

As observed previously [24], the hybrid methods inherit their error range from the underlying lower‐order methods, but the errors are attenuated. MRCC/CEPA(0) gives nearly perfect results for the size‐consistency tests, just as MRCEPA(0), with only a small error for the entangled spin state. For demonstrative purposes, we also formulated a hybrid method from MRCCSD and MRCISD, and the values in Table 3 indeed demonstrate that the size‐consistency errors of MRCISD can be reduced to a third by this approach. For practical purposes, this is still not good enough, and we will not further consider these methods in the remaining part of this work. The reduction of the size‐consistency errors is smaller (typically a reduction to 50 percent) for the other cases and most noticeable in those cases where orbital shells are split among the subsystems [all tests except tests (a) and (b)]. Most disturbing is the still noticeable influence of a non‐interacting Ne atom on the singlet‐triplet splitting [tests (b) and (d)], which are on the order of 0.5 kJ/mol for MRCC/ACPF and MRCC/AQCC for test (d), while better results are here obtained for ACEPA (< 0.1 kJ/mol).

Shifting the single excitations to the coupled‐cluster space (all methods with the suffix‐S in Table 3) has no significant effect on the size‐consistency errors, but in all cases the error is reduced.

In summary, the tests show that the MRACPF, MRAQCC, and MRACEPA introduce significant size‐consistency errors (although less dramatic than for MRCISD), which is clearly a regression compared to MRCEPA(0). This deficiency has to be kept in mind for the overall assessment of the methods.

### 4.2 Potential Energy Curves

4.2.1

The carbon dimer is a prototypical case for a complex electronic structure and has been the subject of many theoretical studies. Just to mention a few, Müller and co‐workers [41] presented one of the first comprehensive studies of the spectroscopic constants of the most important low‐lying states based on uncontracted MRCI and MRAQCC methods, Gulania and co‐workers [42] discussed the computation of these states via equation‐of‐motion double‐ionization coupled‐cluster (EOM‐DIP‐CC) methods, Jiang and Wilson [43] explored multireference composite approaches, and Sharma and Alavi [44] used the ground state potential energy curve as a demonstrator for their matrix‐product‐state implementation of a linearized coupled‐cluster doubles (LCCD) method. The strong multireference character of this molecule originates from a near‐degeneracy of the $2\sigma_{u}$, $3\sigma_{g}$, and $1\pi_{u}$ orbitals at equilibrium distances, and the complexity increases for slightly elongated bond distances due to the breaking of π bonds.

For this study, we use a full valence CAS(8,8) and a cc‐pVTZ basis set and focus on the three states 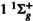, 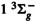 and 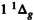. A notable difficulty for the 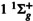 state is an avoided crossing with the 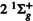 state, which can lead to artifacts in correlation methods that do not properly take into account the near degeneracy effects [45]. Another difficulty is the presence of the 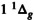 state. In the Abelian subgroup $D_{2h}$, as exploited by most computational packages, one component of 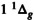 falls into the same irreducible representation as the 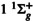 state and requires a careful optimization of the CASSCF orbitals and tracking of the states along the potential energy curve.

Unfortunately, near‐degeneracies pose a problem for the reference relaxation procedure for distances beyond 1.6 Å and probably require an improved multistate approach [46]. Therefore, we will confine ourselves to discussing the results for the computations without reference relaxation; results including reference relaxation for the 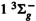 state can be found in the supporting information Figure S1.

**FIGURE 1:**
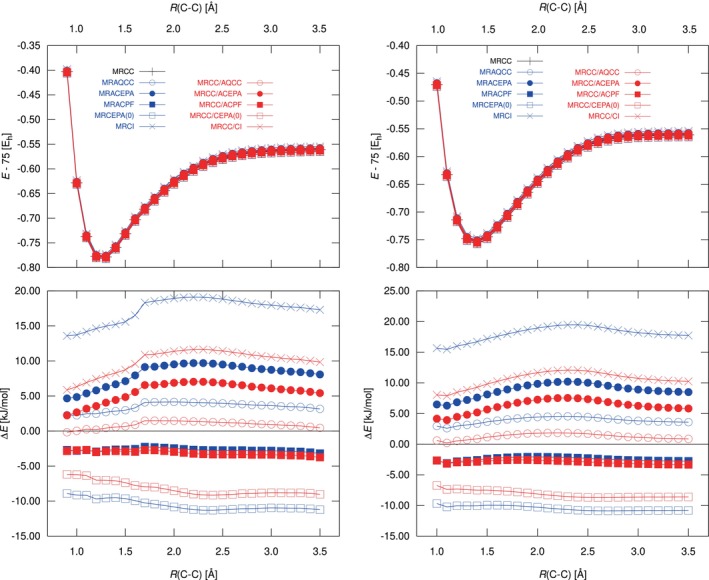
Potential energy curves of 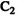 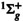 (left) and 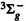 (right). The upper plots show total energies ($E_{\text{h}}$ units), while the lower plots show the deviations from icMRCCSD (in kJ/mol). Reference relaxation was not used for these tests (see text).

In Figure 1 we show the potential energy curves for the lowest 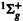 and 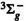 states along with the deviations of the different methods from icMRCCSD. The potential energy curve of the 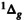 state can be found in the supporting information Figure S2. Note that the main aim of this study is to compare more approximate methods to icMRCCSD in order to assess which of those could be a viable alternative. No comparison to more accurate methods or experimental references is attempted here. Füsti‐Molnár and Szalay [33], Ruttink et al. [21] and Köhn et al. [30] performed such assessments against full Configuration Interaction (FCI) for MRCEPA(0), MRACPF, MRAQCC [5, 33], MRACEPA [21], and MRCCSD [30], respectively. MRACPF and MRAQCC present here a significant reduction in deviation with respect to FCI as compared to MRCEPA(0), and MRACEPA is approximately on a par with the first two. MRCCSD shows relatively low deviations compared to FCI, rendering comparisons to it sufficient in this context. A comparison with respect to the experiment is discussed in Section 4.3.

In general, we find that the curves of all methods run quite parallel with deviations growing towards 2.5 Å and slightly decreasing afterwards. The largest deviation is found for MRCI, which is short of 15–20 kJ/mol in correlation energy in comparison to the coupled‐cluster reference (note that no Davidson correction [47] is considered here). MRCEPA(0) overshoots the correlation energy strongly, leading to deviations of more than −10 kJ/mol, while MRACPF and MRAQCC are rather close to the icMRCCSD method (absolute deviations of less than 5 kJ/mol). MRACPF slightly overshoots but is also very parallel to the reference curve. The more elaborate MRACEPA method, however, deviates more strongly from the reference result; the deviation is more than twice as large as that of MRAQCC. Overall, we see a clear trend that the amount of correlation energy predicted by the different methods correlates with the amount of correlation energy that enters the shift factor. MRCEPA(0) fully ignores the shift and clearly overshoots, while for MRCI the full correlation energy enters, leading to significantly too small correlation energies.

With the hybrid methods, the deviations to icMRCCSD are significantly reduced for most methods; only for MRACPF and MRCC/ACPF are there nearly no differences. The overall best result (in the sense of closely agreeing with icMRCCSD) is obtained for the MRCC/AQCC hybrid.

As a side note, there is a slight discontinuity at 1.7 Å seen in Figure 1 for the differences in the 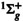 state. The reason for this is likely the mixing of the 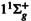 and 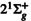 states, as also pointed out by Szalay [45] in a work on an improved (uncontracted) variant of MRAQCC for near‐degenerate states. A detailed analysis of this behavior, in particular in the context of internally contracted methods, is an important issue for our further working agenda but goes beyond the scope of this present work.

For a more quantitative overview, we also report in Table 4 the non‐parallelity errors (NPEs) and mean absolute deviations (MADs) for the ranges plotted in Figure 1; in addition, we also give the values for the 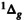 state. The NPE is defined as the difference $\Delta E_{\max}-\Delta E_{\min}$, with $\Delta E_{\max}$ and $\Delta E_{\min}$ being the maximum and minimum deviation with respect to icMRCCSD. The values confirm our observations from above; in particular, the hybrid method MRCC/AQCC performs very well with an MAD of less than 1.3 kJ/mol for all states, and the NPE is less than 1.7 kJ/mol. MRACPF and its hybrid MRCC/ACPF have very similar figures of merit; the MAD (2.5–3.5 kJ/mol for the three states) is slightly larger than for MRCC/AQCC, but their NPE is smaller (around 1 kJ/mol for all three states). The hybrid methods with alternative partitioning, shifting the ${\hat{T}}_{1}$ operators into the coupled‐cluster part, unfortunately turn out to introduce significant instabilities, and for the 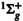 and 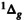 states we were not able to obtain converged results for the MRCC/ACPF‐S and MRCC/AQCC‐S case. For all other cases, the NPE and MADs are given in Table 4, but the numbers show that this variant hardly results in any improvement.

**TABLE 4:** Non‐Parallelity Error (NPE) and Mean Absolute Deviation (MAD) in kJ/mol for three different states of of the respective curves in Figure 1. Reference relaxation was disabled for all calculations.

	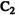 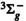		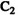 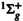		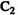 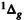	
Method	NPE^a^	MAD^a^	NPE^b^	MAD^b^	NPE^b^	MAD^b^
MRCISD	4.017	18.006	5.517	17.306	3.366	18.007
MRCEPA(0)	1.186	10.457	2.376	10.531	1.631	11.192
MRACPF	1.016	2.470	0.929	2.723	0.777	2.982
MRAQCC	1.940	3.863	1.912	3.469	1.531	3.518
MRACEPA	3.909	8.805	5.035	8.180	3.664	8.507
MRCC/CEPA(0)	1.968	8.151	2.967	8.213	2.406	8.782
MRCC/CEPA(0)‐S	1.957	8.340	3.574	8.171	2.681	8.804
MRCC/ACPF	0.769	2.922	1.050	3.145	0.995	3.437
MRCC/ACPF‐S	1.895	2.079	n.c.^c^	n.c.^c^	n.c.^c^	n.c.^c^
MRCC/AQCC	1.633	1.242	1.656	0.903	1.283	0.813
MRCC/AQCC‐S	1.091	2.038	n.c.^c^	n.c.^c^	n.c.^c^	n.c.^c^
MRCC/ACEPA	3.608	6.233	4.796	5.649	3.885	5.853
MRCC/ACEPA‐S	3.425	5.995	7.047	4.924	5.640	5.481

^a^ Evaluated for a grid with 0.1 Å steps from 1.0 to 3.5 Å.

^b^ Evaluated for a grid with 0.1 Å steps from 0.9 to 3.5 Å.

^c^ Not converged.

#### 4.2.2 N_2_ and CN

Two additional dinuclear compounds were considered to complement the picture. In particular, we wanted to include examples that involve additional excitation classes, as the previous example only involved the $S_{0}$ and $P_{0}$ types of excitations (see Table 1). We used a CAS(6,6) for N_2_ molecule (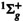 state) such that the $2\sigma_{g}$, and $2\sigma_{u}$ orbitals are left in the closed shell part of the orbitals space but are correlated. For CN (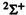 state), a CAS(7,6) is a viable alternative to the full valence CAS(9,8) and is constructed by dropping the 3σ orbital (mostly N 2s character) and the 6σ orbital [48]. In this case, the 3σ orbital is correlated as a closed‐shell orbital, while the 6σ orbital is added to the virtual orbitals. For reference, full valence CAS(9,8) results of CN are shown in the supporting information Figure S3. For N_2_ we employed the def2‐QZVPP basis to allow direct comparison to previous publications [23, 24], while the CN calculations employ the cc‐pVTZ basis set. In these cases, reference relaxation does not pose any problem, and all computations reported here are based on this approach. For comparison, results without reference relaxation can be found in the supporting information Figures S4–S6.

**FIGURE 2:**
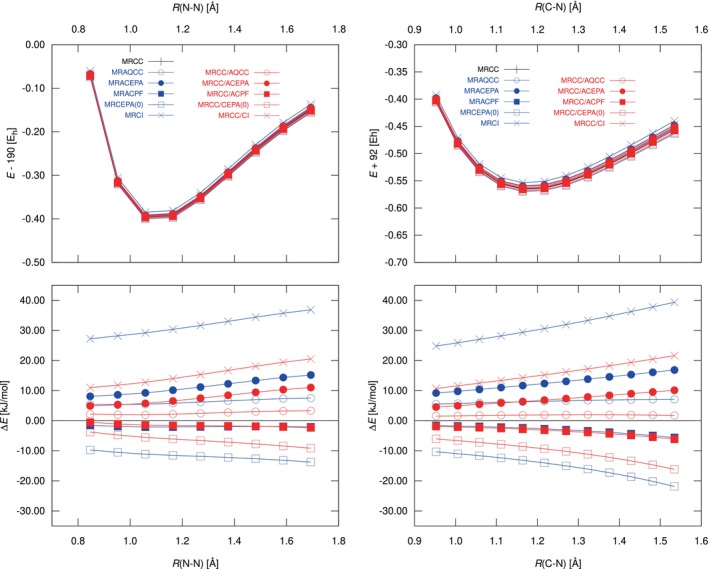
Potential energy curves of N_2_ 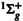 (left) and CN 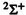 (right). The upper plots show total energies ($E_{\text{h}}$ units), while the lower plots show the deviations from icMRCCSD (in kJ/mol). All computations include reference relaxation.

As shown in Figure 2, the results confirm the findings from the 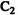 example in the previous paragraph; the main differences are the larger deviations for MRCISD, which have their roots in the presence of correlated electrons outside the active space. The other methods show roughly the same deviations from icMRCCSD as for 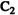; the resulting NPEs and MADs are summarized in Table 5. Interestingly, the results do not indicate any significant advantage of MRACEPA, which chooses different shift factors for the various correlation contributions, over the much simpler MRACPF and MRAQCC methods. As before, MRACPF and the MRCC/ACPF hybrid behave very similarly, while MRAQCC is clearly improved by moving to the MRCC/AQCC hybrid, which confirms the excellent results seen for 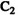.

**TABLE 5:** Non‐Parallelity Error (NPE) and Mean Absolute Deviation (MAD) in kJ/mol for the N_2_ and CN curves in Figure 2. Reference relaxation was used in all cases.

	N_2_ 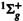		CN 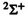	
Method	NPE^a^	MAD^a^	NPE^b^	MAD^b^
MRCISD	9.618	30.491	14.548	31.627
MRCEPA(0)	3.991	11.439	11.527	15.118
MRACPF	0.563	1.988	4.037	3.155
MRAQCC	2.152	5.906	1.548	6.463
MRACEPA	7.087	11.386	7.656	12.852
MRCC/CEPA(0)	5.366	5.985	10.114	10.276
MRCC/ACPF	1.802	1.454	4.238	3.567
MRCC/AQCC	1.338	2.358	0.448	1.825
MRCC/ACEPA	6.094	6.912	5.579	7.205

^a^ Evaluated for a grid with 0.1 $a_{0}$ steps from 1.6–3.2 $a_{0}$.

^b^ Evaluated for a grid with 0.1 $a_{0}$ steps from 1.8–2.9 $a_{0}$.

#### 4.2.3 O_3_

The ozone molecule has, at several instances, been a critical test system for new methods. For example, the importance of the singles projection terms that distinguish the CCSD(T) method [49, 50] from its predecessor CCSD+T(CCSD) [51] (later known as CCSD[T]) was demonstrated for the antisymmetric stretching mode, which is dramatically underestimated by CCSD[T] [52, 53]. On the other hand, the symmetric stretch coordinate of O_3_ revealed critical shortcomings of the MRCEPA(0) method [19] or the second‐order approximate coupled‐cluster method CC2 [54], which both lead to infinitely large correlation energies at stretched geometries, for CC2 even without any barrier [54].

**FIGURE 3:**
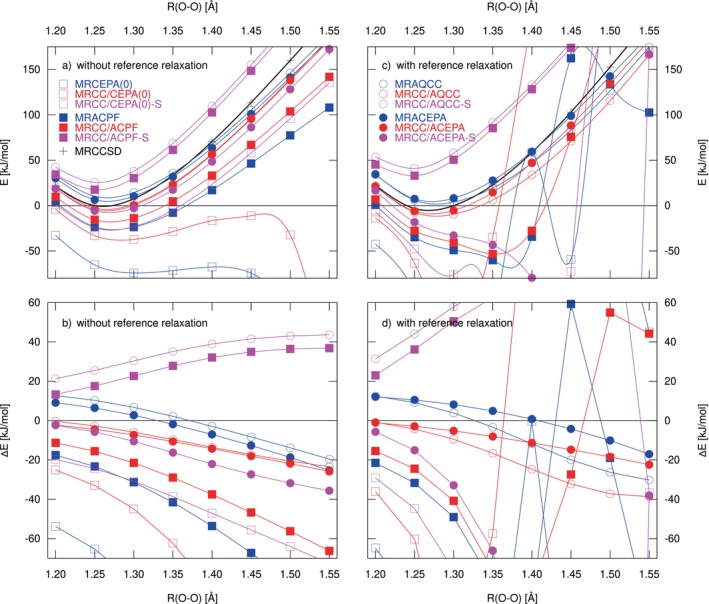
Symmetric stretch of O_3_ using CAS(2,2) references and the cc‐pVTZ basis set. The angle between the oxygen atoms was kept at 116.8°. Panels (a) and (b) show the results from computations without reference relaxation; for (c) and (d), reference relaxation was enabled. The computed points are connected by cubic splines as a guide to the eye. For better comparison, all energies in the upper panels (a) and (c) are given relative to the energy at R(O‐O) = 1.25 Å. The lower panels (b) and (d) show energies relative to icMRCCSD.

In Figure 3 we show cuts of the potential energy surface of O_3_ along the symmetric stretch coordinate for a fixed angle of 116.8°. A minimal active space, CAS(2,2), was used for this set of calculations. Of course, the active space is too small to allow a proper description of the dissociation, but still it is advantageous if a method provides stable solutions under these conditions. Results for CAS(12,9) are shown in the supporting information Figure S7.

Without reference relaxation, most of the methods pass this test in the sense that they provide a monotonously increasing potential energy curve, even for strongly stretched geometries. One of the obvious exceptions is MRCEPA(0), which leads to a significantly too shallow potential energy curve and breaks down for elongations beyond 1.4 Å. This is the behavior also found by Szalay and Bartlett [19]. The hybrid MRCC/CEPA(0) performs slightly better but also breaks down for large elongations. Interestingly, in this case the additional inclusion of non‐linear terms in ${\hat{T}}_{1}$ improves the performance; the hybrid MRCC/CEPA(0)‐S remains stable up to the maximum tested bond distance of 1.55 Å. The deviations from MRCCSD, Figure 3b, however, still indicate a strongly increasing correlation energy. A similar behavior can be found for MRACPF and its hybrid MRCC/ACPF, as well.

When reference relaxation is included, the equations for the latter two methods as well as MRCC/CEPA(0)‐S become unstable, and strongly erratic behavior is found; see Figures 3c and d. In addition, also MRCC/ACEPA‐S shows a diverging correlation energy; interestingly in this case the additional shift of the $T_{1}$ operators into the coupled‐cluster part is detrimental; for the MRACEPA and MRCC/ACEPA, no significant problems are observed. Also, in the case of MRCC/AQCC‐S and MRCC/ACPF‐S, the alternative partitioning scheme does not improve the overall performance. There is no breakdown for larger bond distances, and the equations also stay stable when reference relaxation is included, but the resulting potential energy curves appear too stiff; that is, they show a clearly increased curvature.

For the antisymmetric stretch of O_3_, a less problematic behavior is found. In Table 6 we summarize the frequencies that result from the computed curvatures. These were obtained using 3 distorted configurations along the antisymmetric stretch coordinate (using a bond angle of 116.8° and a bond distance of 1.272 Å as equilibrium geometry). For each distorted geometry, the two bond distances were varied by 0.005 Å, and the second derivative was computed using a seven‐point stencil [55].

**TABLE 6:** Antisymmetric stretch mode of O_3_ in cm^−1^. The cc‐pVTZ basis set was used.

	Relaxed reference	Unrelaxed reference	
Method	CAS(2,2)	CAS(2,2)	CAS(12,9)
MRCCSD	1215	1214	1132
MRCEPA(0)	1039	1070	1131
MRACPF	1154	1150	1129
MRAQCC	1187	1186	1127
MRACEPA	1204	1184	1096
MRCC/CEPA(0)	1023	1140	1131
MRCC/CEPA(0)‐S	1121	1176	1133
MRCC/ACPF	1171	1181	1129
MRCC/ACPF‐S	[2231]	1215	1124
MRCC/AQCC	1196	1199	1128
MRCC/AQCC‐S	1278	1221	1122
MRCC/ACEPA	1194	1185	1121
MRCC/ACEPA‐S	1206	1194	1123

Using a CAS(2,2), the value of 1215 cm^−1^ obtained for MRCCSD is too high in comparison to the experimental reference (1089 cm^−1^, deperturbed harmonic value from Reference [56]) and it is known that triple excitations are required for a more quantitative result [10, 57]. Reference relaxation does not have a significant impact on the frequency; on the other hand, a better agreement with the experiment is obtained with a larger CAS(12,9), see Table 6.

For the small active space, MRCEPA(0) as well as its hybrids result in a significantly weaker antisymmetry potential curve, which in this case leads to fortuitously good agreement with the experimental reference. Among the other methods, MRAQCC performs closest to MRCCSD, in particular as hybrid MRCC/AQCC. The variants with alternative partitioning of the single excitations do in general not improve the performance of their parent method; for MRCC/ACPF‐S the equations get instable when reference relaxation is requested.

For the larger CAS(12,9), only little differences are found between methods. The strongest ‘outlier’ is found for MRACEPA; its value of 1096 is somewhat closer to the experimental reference but clearly farther away from icMRCCSD than all the other approximate methods. All the other results fall into the range of 1121 and 1133 cm^−1^, where the icMRCCSD value is 1132 cm^−1^.

The main lesson from this paragraph is that among all tested approximations to icMRCCSD, only MRAQCC and its hybrid MRCC/AQCC stand out as sufficiently stable methods.

### 4.3 Singlet‐Triplet Splitting of Benzynes

As a last benchmark, we discuss the adiabatic singlet‐triplet splitting of ortho‐, meta‐, and para‐benzene. The benzenes have a singlet ground state with diradical character increasing from ortho to para‐benzene [58]. Along with that, the multireference character of the singlet state increases and the singlet‐triplet splitting decreases.

We use the optimized singlet and triplet geometries from Evangelista and co‐workers [59] and a CAS(2,2) active space. There has been some discussion in the literature [60, 61, 62], to what degree this active space is sufficient, as the π system undergoes spin polarization that leads to a through‐bond coupling of the radical centers [62]. We note that for highly correlated theories like icMRCC, the size of the active space is a much smaller issue as compared to perturbation theory or linear CI theories. In Reference [57] we have demonstrated that if triple excitations are included via a icMRCCSD(T) approach, the same singlet‐triplet gap is predicted for para‐benzyne with both a CAS(2,2) and a CAS(8,8) within 0.2 kcal/mol (9 meV). For approaches limited to pair excitations, an extension of the active space can improve results, and it was shown in Reference [57] that for icMRCCSD the extension from CAS(2,2) to CAS(8,8) adds 39 meV to the gap, while the (T) correction on top of a CAS(2,2) adds 43 meV. We will here follow the philosophy that the active space should remain as small as possible within a coupled‐cluster framework, and our ultimate goal will be to complement our hybrid approaches with a perturbative correction for higher‐order clusters. Therefore, we will here focus on CAS(2,2) computations and the question, which of the approximate methods are viable replacements for icMRCCSD.

An assessment with respect to the experiment is possible by using the difference between band offsets in the photoionization spectra provided by Wenthold et al. in Reference [63]. corrected by the vibrational effects computed by Evangelista and co‐workers in Reference [59]. These results for o‐benzyne show an adiabatic singlet‐triplet splitting of 1641 ± 13 meV, for m‐benzyne to a splitting of 881 ± 14 meV, and for p‐benzyne a splitting of 152 ± 16 meV. These splittings compare well with those obtained for icMRCCSD: We already found in previous studies (References [24] and [29], see also there for a more detailed discussion) 1587 meV for o‐benzyne, 805 meV for m‐benzyne, and 159 meV for p‐benzyne. These values are also listed in Table 7, and we use those as references for the other methods.

**TABLE 7:** Adiabatic singlet‐triplet splitting of benzynes (in meV). The computed splittings are given for MRCCSD; for all other methods, the deviations to MRCCSD are shown.

Method	o‐benzyne	m‐benzyne	p‐benzyne
MRCCSD	1587	805	159
MRCEPA(0)	−51	−59	103
MRACPF	−37	−40	28
MRAQCC	−38	−37	−8
MRACEPA	−63	−51	−11
MRCC/CEPA(0)	−9	−25	103
MRCC/CEPA(0)‐S	2	−21	94
MRCC/ACPF	−9	−14	34
MRCC/ACPF‐S	−7	3	−58
MRCC/AQCC	−20	−17	0
MRCC/AQCC‐S	−19	−2	−69
MRCC/ACEPA	−31	−28	−1
MRCC/ACEPA‐S	−27	−31	29

MRCEPA(0) and the hybrid MRCC/CEPA(0) have already been discussed in our previous work [24]. While the method slightly underestimates the gap for the ortho and meta compound, the gap of para‐benzene is more strongly overestimated, leading also to a rather large relative error due to the small gap. The hybrid methods slightly improve the results for ortho and meta‐benzenes, but not for para‐benzyne.

The methods MRACPF, MRAQCC, and MRACEPA lead to clearly improved results compared to MRCEPA(0), in particular, the error in the gap relative to icMRCCSD is more systematic. The best results are found for MRAQCC, and turning this method into the MRCC/AQCC hybrid method leads to further improvements, which confirms the findings from the previous sections. As before, the “‐S” partitioning scheme does not lead to improvements.

## 5 Conclusions

In this work, we have presented a set of simplified approximations to internally contracted multireference coupled‐cluster theory with single and double excitations (icMRCCSD). These approximations are based on the idea of creating hybrid methods, where numerically demanding parts of icMRCCSD, typically concerned with internal and single excitations (i.e., excitations that do not promote electrons to virtual orbitals or at most one virtual orbital), are treated by a linearized method, whereas the pair excitations are still treated at the coupled‐cluster level. Such an idea has been pursued previously by our group using the MRCEPA(0) approach [17], but the resulting method has weaknesses, such as a strong overestimation of the correlation energy, too weak chemical bonds, and an early breakdown of the method for small active spaces. These weaknesses are inherited from the MRCEPA(0) method and have been previously discussed in the literature [19]. In the present work, we investigated whether methods that improve on MRCEPA(0) by introducing shifts can be used to provide a better hybrid method.

The most promising results were obtained by employing the MRAQCC method [19, 20], which uses a shift that is based on the number of correlated electron pairs [19]. The resulting hybrid method, termed MRCC/AQCC, leads to correlation energies that, for the examples evaluated during this work, match that of icMRCCSD within 1–2 kJ/mol per atom and have rather low non‐parallelity errors, such that deviations for relative energies are even lower. The method shares the stability of coupled‐cluster methods in critical cases like the symmetric stretch of the O_3_ bonds for small active spaces, a test case for which many other alternative options like the MRACPF method lead to problems. The main drawback of replacing MRCEPA(0) with MRAQCC is the introduction of appreciable size‐consistency errors. The size of these errors is an order of magnitude smaller than those observed for uncorrected configuration interaction schemes, but disturbing effects like slight shifts of relative energies depending on the absence or presence of non‐interacting molecules can occur. In comparison to the pure MRAQCC method, the hybridization with MRCCSD clearly improves the accuracy and the size of the consistency errors. The present implementation, however, does not yet provide all features that are available for current implementations of MRAQCC, which *inter alia* also includes analytic gradients [64].

We also tested the MRACEPA method [21] which uses a more sophisticated construction of the energy shift, but in our experience this did not improve the results. The reasons for this behavior have not been analyzed in detail and may be an interesting subject for future work. In addition we tested alternative hybrid methods, which also treated the single excitation cluster operators in the coupled‐cluster part. These tests did not indicate improved results; rather convergence, problems were found in a number of cases.

In summary, the MRAQCC method appears to be a promising improvement for designing a hybrid‐based approximation to icMRCCSD, but the impact of the size‐consistency error has to be watched closely in applications. As an alternative to MRAQCC, more elaborate MRCEPA schemes with electron pair‐dependent shifts [15] are an interesting target for further investigations.

## Supporting information


**Data S1** Supporting Information.

**Data S2** Supporting Information.

## Data Availability

The data that supports the findings of this study are available in the Supporting Information of this article.
