# Supplementary material for: Simplified Multireference Coupled‐Cluster Methods: Hybrid Approaches With Averaged Coupled Pair Theories
Source: J Comput Chem. 2025 Jan 22;46(3):e70020. doi: 10.1002/jcc.70020 (PMC11754727; doi:10.1002/jcc.70020)
Supplement: Supplementary file 1 — Data S1 Supporting Information. [file JCC-46-0-s002.pdf]

Supporting information for:

Simplified multireference coupled-cluster methods: Hybrid approaches with averaged coupled pair theories

Alexander Waigum,* Sarah Suchaneck, and Andreas Köhn†

Institute for Theoretical Chemistry, University of Stuttgart,

Pfaffenwaldring 55, D-70569 Stuttgart, Germany

(Dated: December 6, 2024)

CONTENTS

S1. Additional files 2

S2. Additional Figures for C_2 3

S3. Full valence potential energy curve of CN 5

S4. Potential energy curves of N_2 and CN without reference relaxation 6

S5. Stretch modes of O_3 in different active spaces 9

* koehn@theochem.uni-stuttgart.de

† waigum@theochem.uni-stuttgart.de

S1. ADDITIONAL FILES

This list contains a short explanation of the additional files provided as supporting information. All values computed with reference relaxation have been compared to MRCCSD with reference relaxation, while all values computed without have been compared to MRCCSD without reference relaxation. The files are provided as a ZIP archive (SI-data.zip).

- **Size_Consistency_SI.xlsx**: All values for the size-consistency tests and results of some additional tests using reference relaxation.
- **NPE_MAD_SI.xlsx**: All non parallelity errors and mean average deviations of the potential energy curves grouped together.
- **C2_CAS88_trip_SI.xlsx**: Contains all computed energies needed for the $^3\Sigma_g^-$ state in the CAS(8,8), as well as the energies relative to MRCCSD.
- **C2_sigma_singlet_CAS88_SI.xlsx**: Contains all computed energies needed for the $^1\Sigma_g^+$ state in the CAS(8,8), as well as the energies relative to MRCCSD.
- **C2_delta_singlet_CAS88_SI.xlsx**: Contains all computed energies needed for the $^1\Delta^+$ state in the CAS(8,8), as well as the energies relative to MRCCSD.
- **N2_CAS66_SI.xlsx**: Contains all computed energies needed for the $^1\Sigma_g^+$ state in the in the CAS(6,6), as well as the energies relative to MRCCSD.
- **CN_CAS76_SI.xlsx**: Contains all computed energies needed for the $^2\Sigma^+$ state in the CAS(7,6), as well as the energies relative to MRCCSD.
- **CN_CAS98_SI.xlsx**: Contains all computed energies needed for the $^2\Sigma^+$ state in the CAS(9,8), as well as the energies relative to MRCCSD.
- **O3_SI.xlsx**: Contains all computed energies for the symmetric and antisymmetric distortion of O_3.
- **BENZYNE_SI.xlsx**: Contains all computed energies for o-benzyne, m-benzyne and p-benzyne for their singlet and triplet states as well as the splittings and relative splittings with respect to MRCCSD.

S2. ADDITIONAL FIGURES FOR C_2

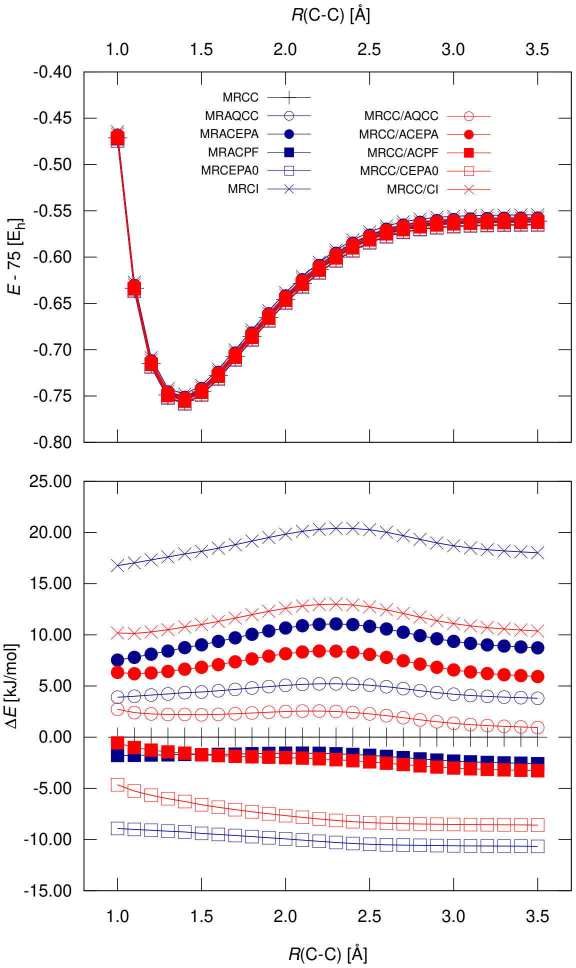

FIG. S1. Potential energy curves of C_2 $^3\Sigma_g^-$ in a CAS(8,8) active space. The upper plot shows total energies (E_h units), while the lower plot shows the deviations from icMRCCSD (in kJ/mol). All results shown **include** reference relaxation.

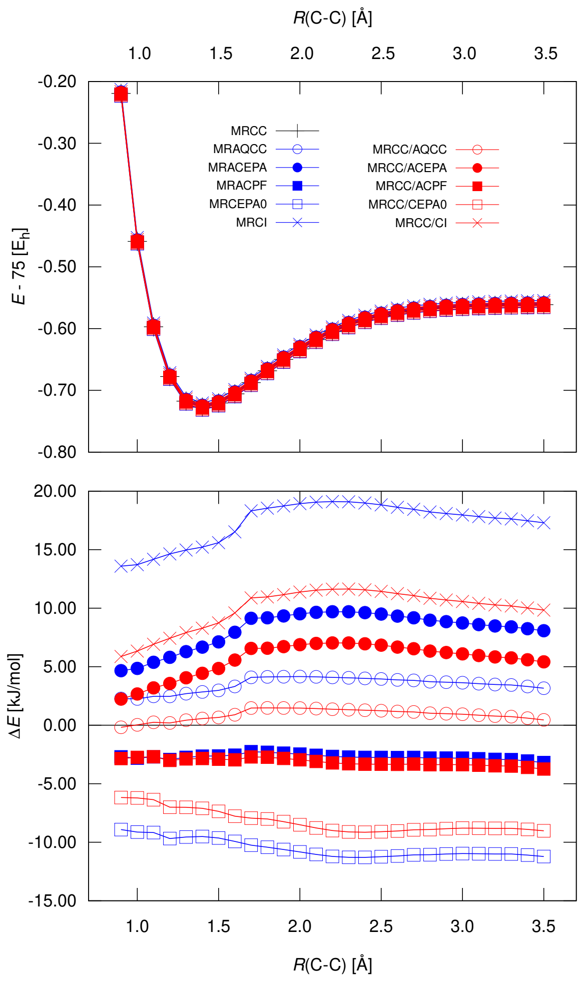

FIG. S2. Potential energy curves of C_2 $^1\Delta_g$ in a CAS(8,8) active space. The upper plot shows total energies (E_h units), while the lower plot shows the deviations from icMRCCSD (in kJ/mol). All results shown **do not include** reference relaxation.

S3. FULL VALENCE POTENTIAL ENERGY CURVE OF CN

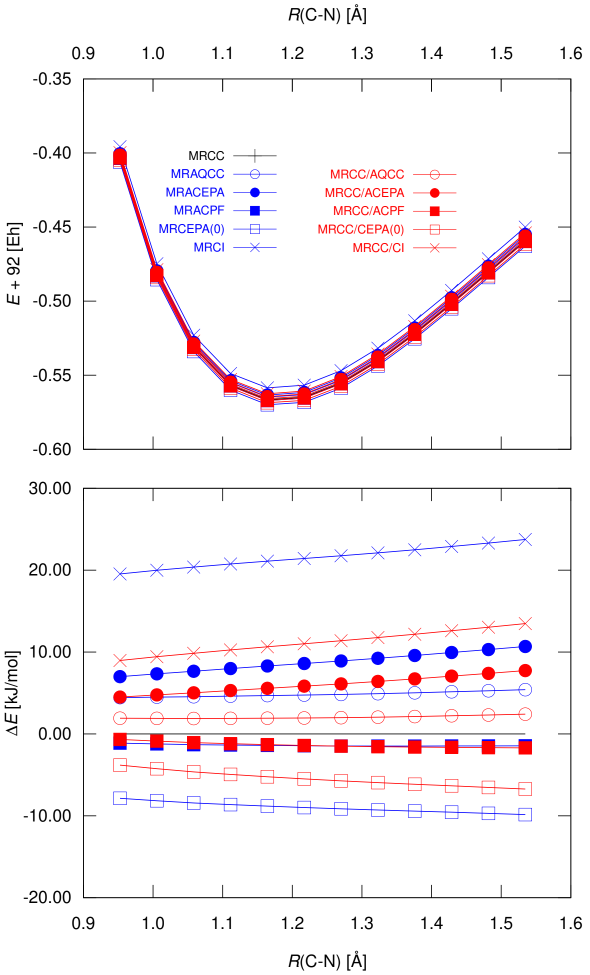

FIG. S3. Potential energy curves of CN $^2\Sigma^+$ in a CAS(9,8) active space. The upper plot shows total energies (E_h units), while the lower plots show the deviations from icMRCCSD (in kJ/mol). All results shown included reference relaxation.

S4. POTENTIAL ENERGY CURVES OF N_2 AND CN WITHOUT REFERENCE RELAXATION

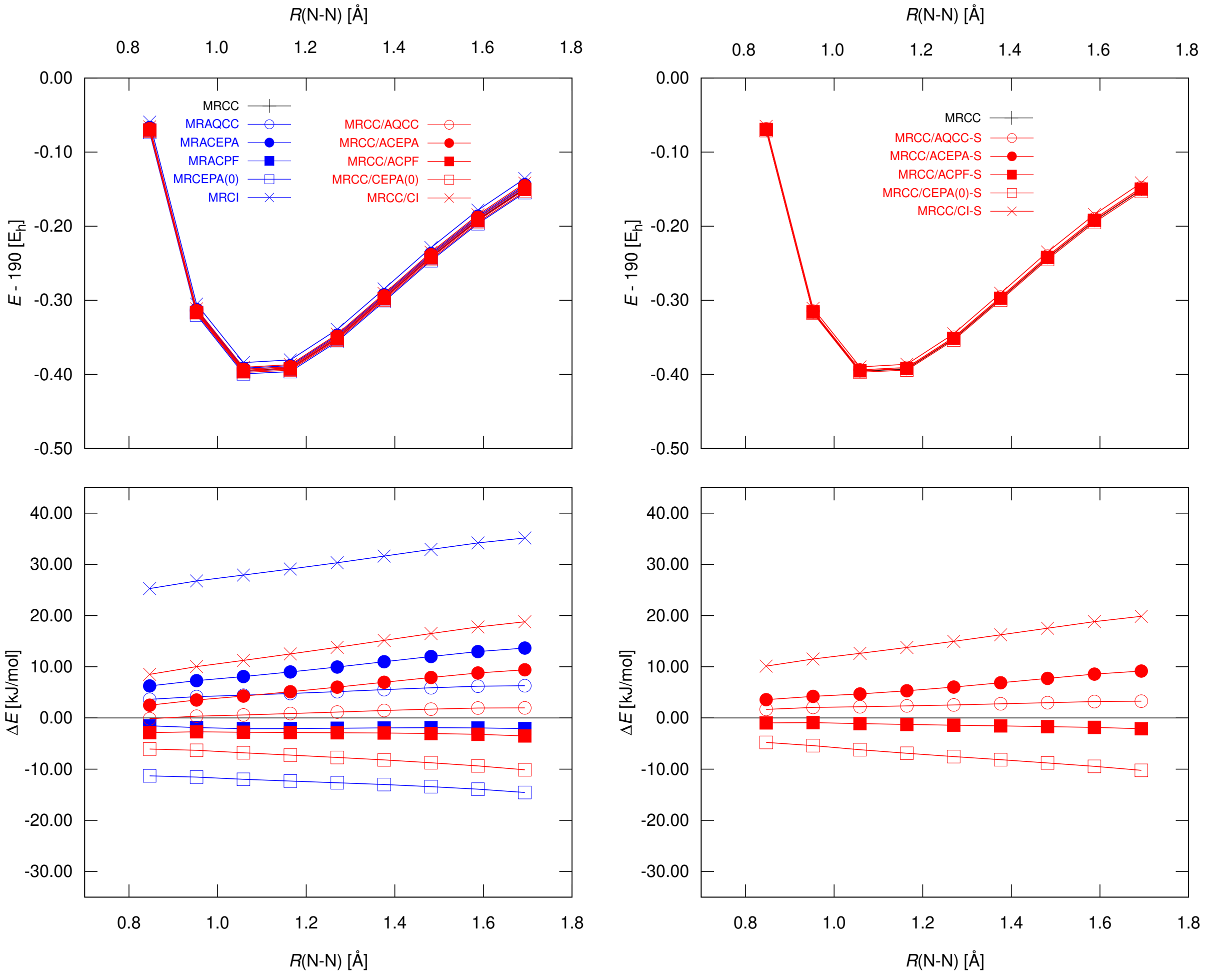

FIG. S4. Potential energy curves of N_2 $^1\Sigma^+$ in a CAS(6,6) active space. The upper plots show total energies (E_h units), while the lower plots show the deviations from icMRCCSD (in kJ/mol). All results shown do not include reference relaxation.

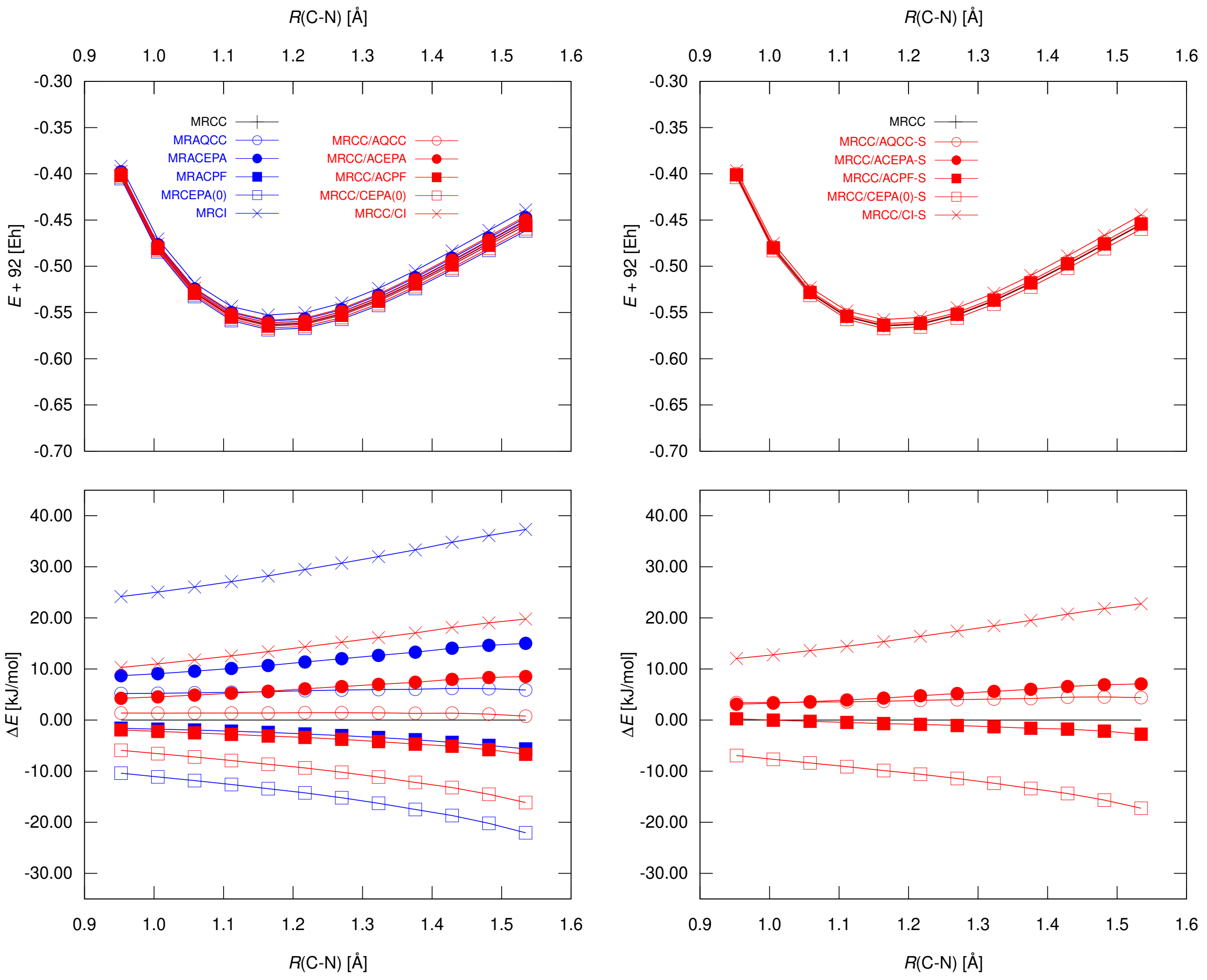

FIG. S5. Potential energy curves of CN $^2\Sigma^+$ in a CAS(7,6) active space. The upper plots show total energies (E_h units), while the lower plots show the deviations from icMRCCSD (in kJ/mol). All results shown do not include reference relaxation.

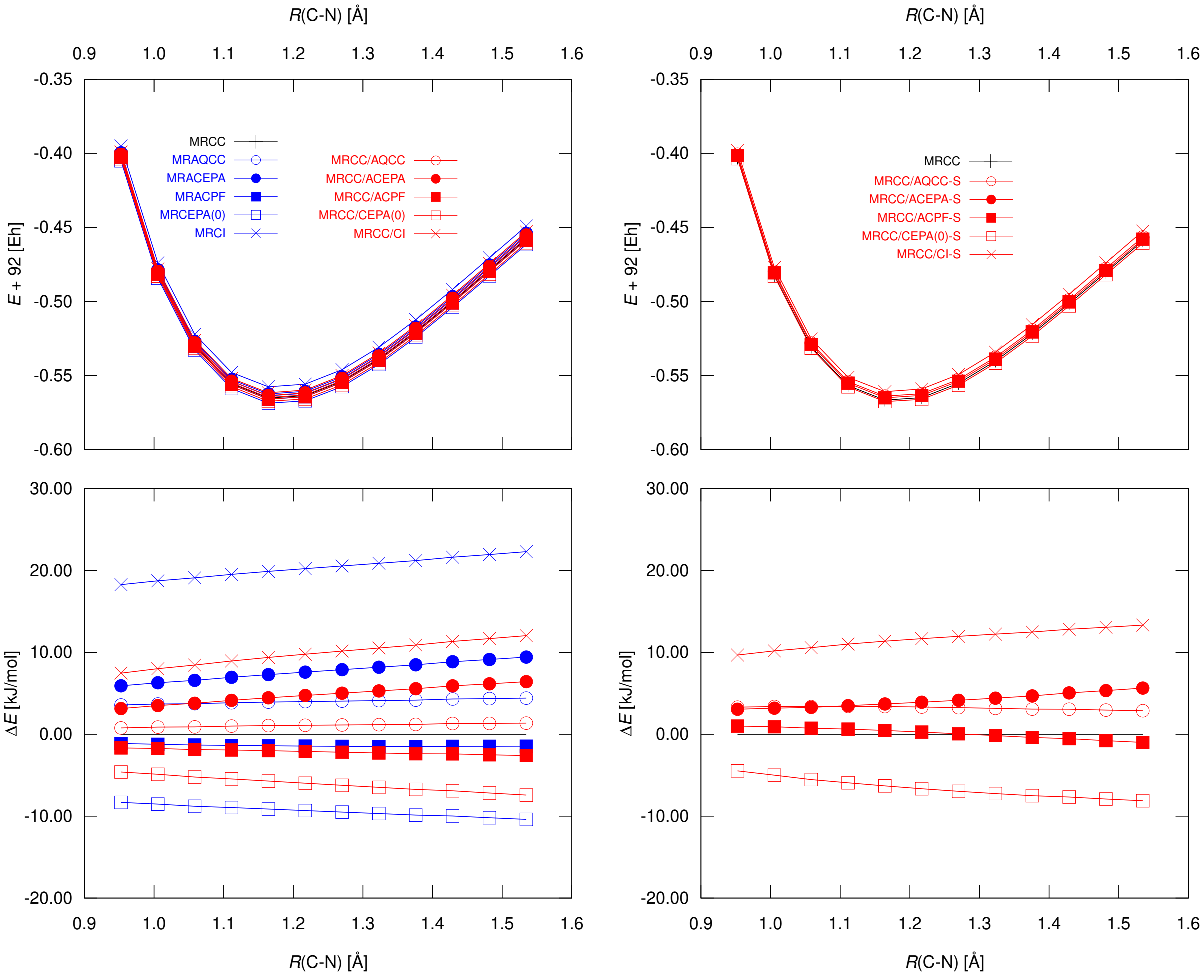

FIG. S6. Potential energy curves of CN $^2\Sigma^+$ in a CAS(9,8) active space. The upper plots show total energies (E_h units), while the lower plots show the deviations from icMRCCSD (in kJ/mol). All results shown do not include reference relaxation.

S5. STRETCH MODES OF O_3 IN DIFFERENT ACTIVE SPACES

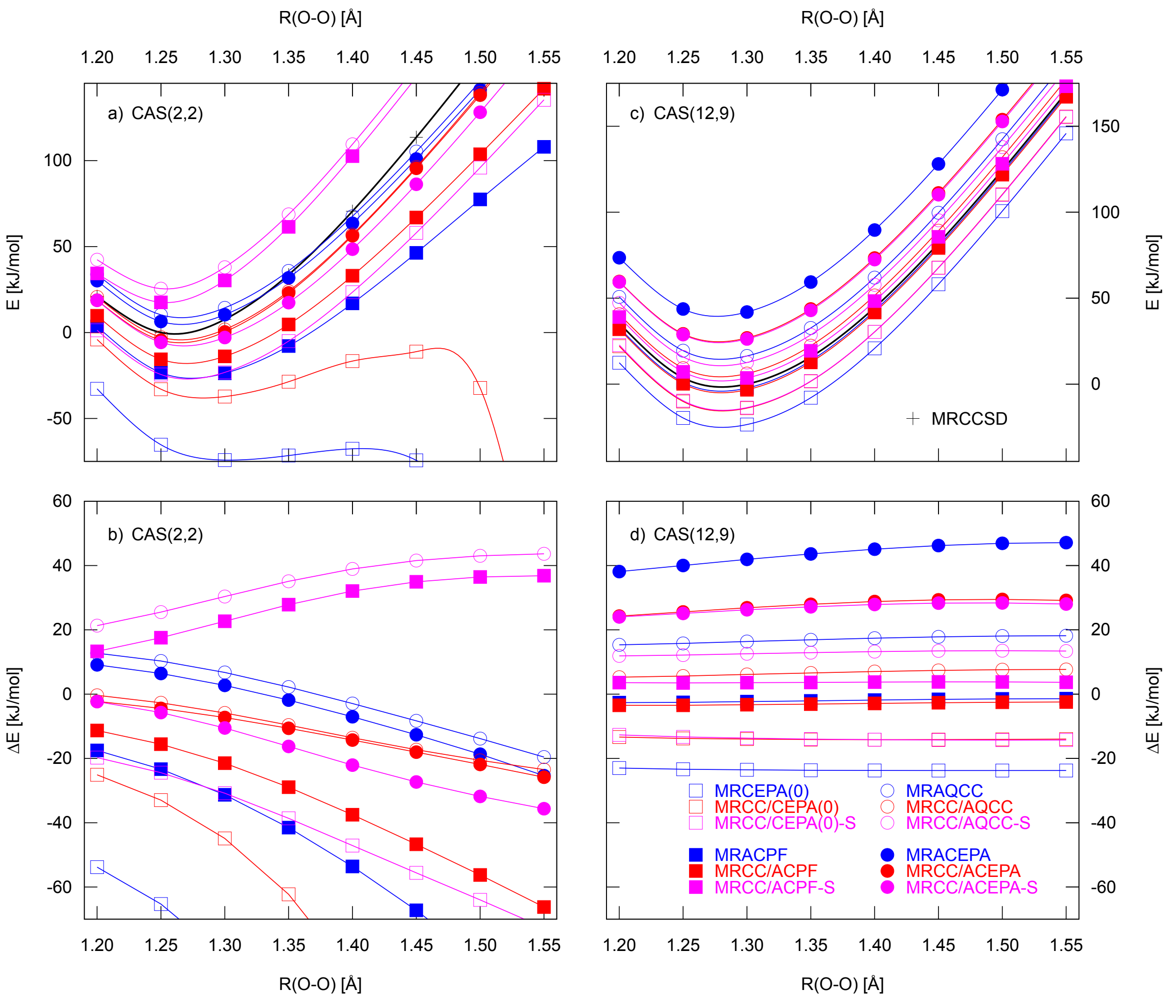

FIG. S7. Comparison of O_3 CAS(2,2) and CAS(12,9), both without reference relaxation.
